# Ten‐Year Post‐traumatic Osteoarthritis Incidence Is Low After Pediatric Isolated Meniscal Surgery but Lower After Meniscal Repair Than Meniscectomy

**DOI:** 10.1002/ars2.70067

**Published:** 2026-08-30

**Authors:** Annika N. Hiredesai, Alejandro M. Holle, Jay R. Patel, Katelyn T. Koschmeder, Sailesh V. Tummala, Jeffrey M. Vaughn, Karan A. Patel

**Affiliations:** ^1^ Mayo Clinic Alix School of Medicine Phoenix Arizona U.S.A.; ^2^ Department of Orthopedic Surgery Mayo Clinic Phoenix Arizona U.S.A.; ^3^ Department of Orthopedic Surgery Phoenix Children's Hospital Phoenix Arizona U.S.A.

## Abstract

**Purpose:**

To examine the 5‐ and 10‐year incidence of post‐traumatic osteoarthritis (PTOA) after isolated, primary meniscal surgery differentiating between meniscal repair and meniscectomy, in pediatric patients.

**Methods:**

Pediatric patients (≤16 years) undergoing primary meniscectomy or meniscal repair (2010‐2023) with ≥5‐year follow‐up were identified in the PearlDiver database. Exclusion criteria included prior or concomitant knee injuries, surgeries, or syndromes. Delayed surgery was defined as ≥90 days after diagnosis. PTOA diagnosis was defined by billing codes. Motion restoration procedures (MRPs) before PTOA diagnosis were recorded. Multivariable logistic and Cox regression were used to assess risk factors for PTOA at 5 and 10 years.

**Results:**

A total of 7555 meniscectomy (mean age 14.7, 50% women) and 2797 meniscal repair (mean age 14.4 years, 46% women) patients met inclusion criteria. At 5 years, PTOA occurred in 2.2% of meniscectomy and 1.4% of repair patients. At 10 years, rates were 2.3% and 1.5%, respectively. Average time to PTOA was 737 days (meniscectomy) and 862 days (repair). When disaggregated by sex, there was no significant difference in sex makeup of those who did and did not develop PTOA (*P* = .430). In the meniscectomy group, risk factors for 10‐year PTOA included MRP (hazard ratio [HR] 6.28, *P* < .001), age ≥12 at surgery (HR 4.84, *P* = .027), and obesity (HR 1.84, *P* < .001). In the repair group, MRPs (HR 5.23, *P* = .023) and depression (HR 2.02, *P* = .027) were significant. These same risk factors were identified for 5‐year PTOA.

**Conclusions:**

The 10‐year incidence of PTOA was 2.2% after meniscectomy and 1.5% after meniscal repair in pediatric patients. MRPs were a consistent risk factor. Age ≥12 and obesity were associated with PTOA after meniscectomy. Depression was associated with PTOA after repair.

**Level of Evidence:**

Level III, retrospective cohort study.

## Graphical abstract

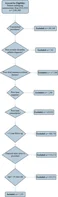

The menisci are essential fibrocartilaginous structures within the knee joint that play a crucial role in joint stability, shock absorption, and load distribution.^1^ Trauma to the menisci and unsuccessful surgical repair of the menisci are both factors that are known to be associated with numerous long‐term complications, including the risk of developing post‐traumatic osteoarthritis (PTOA).^1^ PTOA is a degenerative joint disease that is characterized by a narrowing of the joint space after the breakdown of cartilage triggered by trauma. There is growing evidence suggesting that the type of surgical technique used to repair a meniscus injury can contribute towards the risk of developing PTOA. Current literature reports that the rate of PTOA development in adult patients after a meniscectomy is 17% to 51.4%, and the rate of PTOA development in adult patients after a meniscal repair was 10% to 21.3%.^2^, ^3^ Even a relatively small percentage of meniscal removal can lead to significantly increased tibiofemoral contact pressures.^4^ A 5.7‐fold increased risk of knee osteoarthritis (OA) has been reported in individuals with a history of knee injury compared with uninjured controls over 11 years.^5^

Although meniscal tear type and location are considerations when deciding between meniscal repair and meniscectomy for adults, meniscal repair is increasingly preferred because of concern for poor long‐term outcomes.^6^, ^7^, ^8^ However, although PTOA incidence after meniscal injury is well documented in the adult population, there is limited evidence on PTOA incidence after isolated meniscal injury—and variations by surgical management—in the pediatric population. Although isolated meniscal injury is relatively less common in skeletally immature patients, this injury pattern is becoming more prevalent with increased youth sports participation.^9^ The purpose of this study was to examine the 5‐ and 10‐year incidence of PTOA after isolated, primary meniscal surgery in pediatric patients differentiating between meniscal repair and meniscectomy. We hypothesized that pediatric patients who undergo meniscectomy for isolated, primary meniscal injury will exhibit a higher 10‐year incidence of PTOA compared with those treated with meniscal repair and that distinct patient‐ and procedure‐related factors will independently predict the development of PTOA after meniscal surgery.

## METHODS

### Data Source

Data for this study were obtained from the M170 PearlDiver database, a large, national administrative claims dataset, encompassing over 170 million patients across the United States from 2010 to the third quarter of 2023. The database allows for longitudinal tracking of procedures and complications using International Classification of Disease (ICD) 9^th^ (ICD‐9) and 10^th^ (ICD‐10) revision codes. As the database contains only deidentified, population‐level information, institutional review board approval was not required.

### Patient Selection

A retrospective cohort study was conducted to determine the incidence and risk factors of PTOA after isolated meniscal procedures in pediatric patients. Pediatric patients 16 years of age or younger who underwent isolated meniscal procedures, either meniscal repair or partial meniscectomy, were identified using Current Procedural Terminology codes. Exclusion criteria included tibial eminence avulsion fractures, congenital/syndromic meniscus absence syndrome, juvenile idiopathic arthritis, previous knee OA diagnoses, previous knee injuries/surgeries, and concomitant knee surgeries. This approach aimed to isolate outcomes specifically attributable to meniscal intervention. To ensure appropriate long‐term follow‐up, only patients with continuous enrollment in the database for at least 5 years after the index procedure were included.

### Data Extraction

At the time of the index procedure, demographic and clinical variables were collected, including patient age, sex, depression diagnosis, and obesity status, defined as a body mass index (BMI) over 30. To evaluate if delayed treatment was a risk factor for PTOA development, patients with delayed surgery, defined as surgery beyond 90 days from initial meniscus injury, were identified. Additionally, patients who underwent subsequent motion restoration procedures, including manipulation under anesthesia or lysis of adhesions, were identified.

### Outcome Measures

The primary outcome was the incidence of PTOA of the knee within 5 and 10 years of the index meniscal procedure. PTOA diagnoses were identified using ICD‐9 and ICD‐10 codes for knee OA, recorded at any time point within 10 years after the initial surgery. Notably, ICD‐9 codes do not specifically distinguish PTOA from other forms of OA, so general OA‐related codes were used during the ICD‐9 era (2010‐2015). In contrast, ICD‐10 codes allow for more granular identification and were used to specifically capture PTOA of the knee where available.

### Statistical Analysis

Baseline characteristics were compared using *χ* ^2^ tests for categorical variables and independent t‐tests for continuous variables. Time‐to‐event analysis was conducted to assess the risk of developing PTOA over a 10‐year follow‐up period. Cox proportional hazard regression was used to estimate hazard ratios (HRs) and 95% confidence intervals (CIs), adjusting for potential confounders including age group, sex, obesity status, procedure type, depression diagnosis, delayed surgery, and presence of postoperative motion restoration procedures. The proportional hazard assumption was evaluated using Schoenfeld residuals. No significant time‐dependent effects were observed for any covariate, indicating that the assumption was met, and time‐varying modeling was not required. Subanalyses were performed for procedure type (meniscectomy vs meniscal repair) to identify risk factors specific to each procedure. All statistical analyses were performed using R software provided within the PearlDiver database. Statistical significance was set at a *P* value of .05.

### Ethical Approval

Ethical approval from our institutional review board was waived because all data used in this study was deidentified.

## RESULTS

### Cohort Characteristics

**FIGURE 1:**
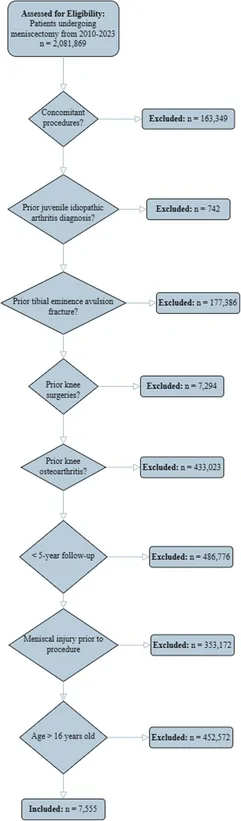
STROBE flowchart for inclusion and exclusion of meniscectomy patients. (STROBE, strengthening the reporting of observational studies in epidemiology.)

**FIGURE 2:**
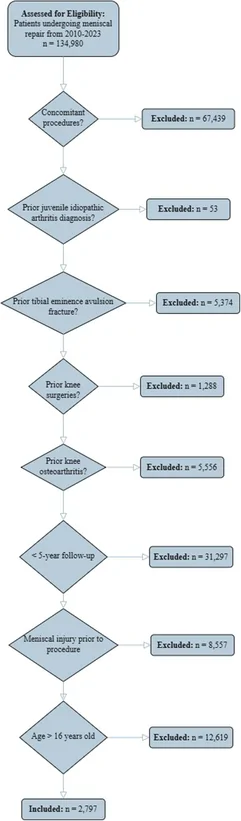
STROBE flowchart for inclusion and exclusion of meniscal repair patients. (STROBE, strengthening the reporting of observational studies in epidemiology.)

There was a total of 10,352 patients who underwent meniscus surgery and met inclusion criteria. Of those, 7555 (73%) underwent meniscectomy (Figure 1) and 2797 (27%) underwent meniscal repair (Figure 2). The cohort had an average age of 14.6 (1.8) and consisted of 5041 (49%) female patients. There were 2358 (23%) patients with a diagnosis of obesity (BMI > 30) and 2701 (26%) with a diagnosis of depression at the time of surgery. A total of 626 (6%) of patients had delayed surgery, with their initial meniscal injury occurring longer than 90 days prior to the index operation. Seventy‐one (0.7%) patients underwent subsequent motion restoration procedures (Table 1).

**TABLE 1:** Demographic Variables and Baseline Characteristics Compared Between Patients With and Without PTOA Diagnosis After Meniscus Surgery

**Variable**	**Total (10,352)**	**No PTOA (10,136)**	**PTOA (216)**	***P*** **Value**
Age	14.6 (1.8)	14.6 (1.8)	15.0 (1.2)	**<.001**
Sex, female	5041 (48.7)	4931 (48.6)	110 (50.9)	.430
Meniscal repair	2797 (27.0)	2754 (27.2)	43 (19.9)	**.013**
Obesity	2358 (22.8)	2285 (22.5)	73 (33.8)	**.001**
Depression	2701 (26.1)	2635 (26.0)	66 (30.6)	.230
Delayed surgery	626 (6.0)	611 (6.0)	15 (6.9)	.970
Subsequent motion restoration procedure	71 (0.7)	63 (0.6)	8 (3.7)	**<.001**

*Note*: Bold *P* values indicate statistical significance set at .05.

PTOA, post‐traumatic osteoarthritis.

There were a total of 201 (1.9%) patients who developed PTOA within 5 years and 216 (2.1%) within 10 years. When compared with patients without PTOA, those who developed PTOA were older (15 vs 14.6 years, *P* < .001), less likely to have undergone a meniscal repair (20% vs 27.2%, *P* = .013), more likely to have obesity at the time of surgery (34% vs 23%, *P* = .001), and more likely to have undergone a subsequent manipulation under anesthesia or lysis of adhesion (3.7% vs 0.6%, *P* < .001). There were no differences in sex, depression diagnosis, or rates of delayed surgery between groups (Table 1).

### Any Meniscal Surgery

On Cox regression, risk factors for PTOA within 10 years after meniscus surgery included subsequent motion restoration procedures (HR, 5.85 [95% CI, 2.74‐12.45]; *P* < .001), age ≥12 years at the time of meniscectomy (HR, 3.88 [95% CI, 1.23‐12.12]; *P* = .020), and obesity (HR, 1.66 [95% CI, 1.23‐2.24]; *P* < .001) (Table 2). These same risk factors were associated with 5‐year PTOA incidence. However, meniscal repair was associated with a decreased rate of PTOA at 5 years (HR, 0.66 [95% CI, 0.47‐0.94]; *P* = .021), but not at 10 years (HR, 0.74 [95% CI, 0.53‐1.03]; *P* = .071).

**TABLE 2:** Risk Factors Associated With Increased Hazard of PTOA Diagnosis 10 Years After Meniscus Procedure

**Risk Factor**	**HR**	**95% CI**	***P*** **Value**
Age ≥12 years	4.06	1.30, 12.70	**.016**
Delayed procedure ≥90 days	1.00	0.54, 1.84	.997
Subsequent motion restoration procedure	5.67	2.66, 12.07	**<.001**
Obesity	1.7	1.27, 2.28	**<.001**
Male sex	0.9	0.68, 1.19	.456
Depression	1.05	0.77, 1.43	.758

*Note*: Bold *P* values indicate statistical significance set at .05.

CI, confidence interval; HR, hazard ratio; PTOA, post‐traumatic osteoarthritis.

### Meniscectomy

There were 173 (2.2%) patients who developed PTOA within 10 years after meniscectomy (Table 3). Cox proportional hazard regression analysis revealed that subsequent motion restoration procedures (HR, 6.28 [95% CI, 2.77‐14.10]; *P* < .001), age ≥12 years at the time of meniscectomy (HR, 4.84 [95% CI, 1.20‐19.61]; *P* = .027), and obesity (HR, 1.84 [95% CI, 1.35‐2.52]; *P* < .001) were independent risk factors associated with PTOA diagnosis 10 years after meniscectomy. These same risk factors were associated with 5‐year PTOA incidence postmeniscectomy.

**TABLE 3:** Risk Factors Associated With Increased Hazard of PTOA Diagnosis 10 Years After Meniscectomy

**Risk Factor**	**HR**	**95% CI**	***P*** **Value**
Age ≥12 years	4.84	1.20, 19.52	**.027**
Delayed meniscectomy ≥90 days	0.94	0.52, 1.69	.830
Subsequent motion restoration procedure	6.28	2.78, 14.19	**<.001**
Obesity	1.85	1.35, 2.52	**<.001**
Male sex	0.91	0.67, 1.23	.528
Depression	0.96	0.68, 1.35	.796

*Note*: Bold *P* values indicate statistical significance set at .05.

CI, confidence interval; HR, hazard ratio; PTOA, post‐traumatic osteoarthritis.

### Meniscal Repair

There were 43 (1.5%) patients who developed PTOA within 10 years after meniscal repair (Table 4). For the meniscal repair cohort, subsequent motion restoration procedures (HR, 5.23 [95% CI, 1.25‐21.82]; *P* = .023) and depression (HR, 2.02 [95% CI, 1.08‐3.77]; *P* = .027) were associated with 10‐year PTOA diagnosis. These same risk factors were associated with 5‐year PTOA incidence after meniscal repair.

**TABLE 4:** Risk Factors Associated With Increased Hazard of PTOA Diagnosis 10 Years After Meniscal Repair

**Risk Factor**	**HR**	**95% CI**	***P*** **Value**
Age ≥12 years	3.56	0.49, 26.06	.211
Delayed meniscal repair ≥90 days	1.72	0.53, 5.61	.365
Subsequent motion restoration procedure	5.23	1.25, 21.82	**.023**
Obesity	1.05	0.50, 2.22	.890
Male sex	1.11	0.89, 0.60	.733
Depression	2.02	0.49, 1.08	**.027**

*Note*: Bold *P* values indicate statistical significance (*P* ≤ .05).

CI, confidence interval; HR, hazard ratio; PTOA, post‐traumatic osteoarthritis.

## DISCUSSION

The main finding of the present study was that the 10‐year incidence of PTOA in the pediatric patients ≤16 years old undergoing isolated, primary meniscectomy or meniscal repair was 2.2% and 1.5%, respectively. Additionally, subsequent motion restoration procedures, age ≥12 years at the time of meniscectomy, and obesity were independent risk factors for PTOA development after meniscectomy. Subsequent motion restoration procedures and depression were independent risk factors for PTOA development after meniscal repair.

There is limited prior evidence on the incidence of PTOA within 5 and 10 years after isolated, primary meniscectomy or meniscal repair in the pediatric population. In a case series of outcomes after primary isolated meniscectomy in pediatric patients, 80% of patients were found to have radiographic changes at average 5.5‐year follow‐up though these changes did not have an effect on clinical outcomes.^10^ However, this study only evaluated total and partial meniscectomy patients. Similarly, Hagmeijer et al. assessed a case series of 44 pediatric patients undergoing meniscal repair for isolated meniscal tear with mean follow‐up of 17.6 years and found excellent long‐term clinical outcomes but did not assess development of arthritis clinically or radiographically.^11^ The rates of PTOA development identified in the present study were consistent with prior literature regarding PTOA development after primary anterior cruciate ligament repair in the pediatric population which identified a 5‐year incidence of 1.6%.^12^ Relative to the well‐documented 10‐year incidence in the adult population, ranging from 16.4% to 23% after meniscectomy and 10% after meniscal repair, PTOA development in the decade after surgery in pediatric patients was notably lower.

There are several key factors that may contribute to skeletally immature patients having lower PTOA incidence after isolated meniscal injury compared with adults. First, pediatric patients are less likely to have pre‐existing chondral wear or OA at the time of injury relative to adults. Furthermore, pediatric meniscal tissue intrinsically has greater healing potential because of enhanced vascularity and cellular activity, making it more amenable to primary repair which has been shown to be preferable to meniscectomy for OA prevention in the adult population.^9^ The tear type also influences healing potential and possibility of meniscal repair, with skeletally immature patients more likely to experience acute peripheral or longitudinal tears versus chronic, degenerative tears in adults.^9^ The combination of robust healing potential and severe consequences of OA development and progression in pediatric patients makes surgeons more aggressive in opting for meniscal repair. The 10‐year rate of PTOA after meniscal repair, while overall small, was significantly lower than in patients undergoing meniscectomy in this present study of skeletally immature patients.^13^ This evidence contributes long‐term data to the current research consensus that, when amenable, meniscal repair is preferable to meniscectomy in pediatric patients.^14^

Subsequent motion restoration procedures, age ≥12 years at the time of surgery, and obesity were all independently associated with both 5‐ and 10‐year PTOA incidence after meniscectomy. All 3 risk factors are consistent with prior literature that considered risk factors for 5‐year PTOA incidence after anterior cruciate ligament reconstruction in the pediatric population.^12^ Subsequent motion restoration procedures are indicated when treating postoperative arthrofibrosis. The combination of persistent synovial inflammation, mechanical trauma during motion restoration, delayed rehabilitation and return to activity, and altered underlying joint kinematics may contribute to the observed increased risk of PTOA development in the pediatric patient.^1^, ^15^ The influence of age on PTOA risk is similarly multifactorial. Prior literature has shown that by the age of 10 years, pediatric menisci have transitioned from highly cellular, fibroblast‐rich structures to a primarily collagen fiber composition.^16^, ^17^ Moreover, the vascularity of the menisci diminishes with skeletal maturity to primarily a peripheral blood supply by 10 years of age as well.^16^, ^17^, ^18^ In a retrospective review of pediatric patients undergoing meniscal surgery, those with closed growth plates were more likely to have complex tears and have higher BMIs.^13^ Moreover, higher BMI was associated with higher risk of complex tear pattern in this same review.^13^ This association between higher BMI and complex tear patterns, which have been associated with poor outcomes, may have contributed to the association between obesity and PTOA risk identified in the present study.^19^

In meniscal repair patients, subsequent motion restoration procedures were similarly associated with increased risk of PTOA, likely because of the same confluence of factors described previously for meniscectomy patients.^1^, ^15^ Depression was a PTOA risk factor unique to patients undergoing meniscal repair in this study. In the setting of anterior cruciate ligament injury, prior literature has linked depression to lower return‐to‐sport rates and adherence to postoperative rehabilitation protocols.^20^, ^21^ Postoperative rehabilitation is particularly crucial in the setting of meniscal repair to protect the repair construct and allow for sufficient biological healing before lifting activity restrictions. Although the present study did not assess return‐to‐sport rates, the association between depression and pain catastrophizing leading to diminished effort and adherence to rehabilitation may be driving this association with increased risk of PTOA in the pediatric meniscal repair population.^22^ These results suggest that depression, as with other orthopaedic procedures, may be a modifiable risk factor for diminished postoperative outcomes. Addressing psychological comorbidities in this patient population may enhance outcomes, especially as the incidence of depression continues to rise in the pediatric population.^23^ The potential benefit of involving resources such as sports psychologists also warrants consideration, particularly in settings where these specialists are available to support patients and their families.^24^, ^25^

### Limitations

The results of this study should be interpreted in the context of the following limitations. As with all large claims databases, coding and billing accuracy was an inherent limitation to the study methods. Radiographs or other advanced imaging studies or reports were unavailable to characterize meniscal tear type and surgical outcomes. Similarly, identification of osteoarthritic changes or grading by Kellgren‐Lawrence scores was also not possible. Variations in rehabilitation duration, protocols, and adherence could not be assessed directly. Patient‐reported outcome measures were also not available for query. PTOA diagnosed in patients from 2010 to 2015 in this study was defined using ICD codes that were not necessarily specific for arthritis caused by trauma because ICD‐9 coding did not make this distinction. Additionally, discoid meniscal pathology could not be identified in this database.

## CONCLUSIONS

The 10‐year incidence of PTOA was 2.2% after meniscectomy and 1.5% after meniscal repair in pediatric patients. Motion restoration procedures were a consistent risk factor. Age ≥12 and obesity were associated with PTOA after meniscectomy. Depression was associated with PTOA after repair.

## DISCLOSURES

The authors (A.N.H., A.M.H., J.R.P., K.T.K., S.V.T., J.M.V., K.A.P.) declare that they have no known competing financial interests or personal relationships that could have appeared to influence the work reported in this article.
